# Transfer of Motor Learning Is More Pronounced in Proximal Compared to Distal Effectors in Upper Extremities

**DOI:** 10.3389/fpsyg.2017.01530

**Published:** 2017-09-08

**Authors:** Tore K. Aune, Morten A. Aune, Rolf P. Ingvaldsen, Beatrix Vereijken

**Affiliations:** ^1^Department of Sport Science and Physical Education, Nord University Levanger, Norway; ^2^Department of Neuromedicine and Movement Science, Norwegian University of Science and Technology Trondheim, Norway

**Keywords:** transfer of learning, specificity of learning, interhemispheric communication, neural inhibition and excitation, skill acquisition

## Abstract

The current experiment investigated generalizability of motor learning in proximal versus distal effectors in upper extremities. Twenty-eight participants were divided into three groups: training proximal effectors, training distal effectors, and no training control group (CG). Performance was tested pre- and post-training for specific learning and three learning transfer conditions: (1) bilateral learning transfer between homologous effectors, (2) lateral learning transfer between non-homologous effectors, and (3) bilateral learning transfer between non-homologous effectors. With respect to specific learning, both training groups showed significant, similar improvement for the trained proximal and distal effectors, respectively. In addition, there was significant learning transfer to all three transfer conditions, except for bilateral learning transfer between non-homologous effectors for the distal training group. Interestingly, the proximal training group showed significantly larger learning transfer to other effectors compared to the distal training group. The CG did not show significant improvements from pre- to post-test. These results show that learning is partly effector independent and generalizable to different effectors, even though transfer is suboptimal compared to specific learning. Furthermore, there is a proximal-distal gradient in generalizability, in that learning transfer from trained proximal effectors is larger than from trained distal effectors, which is consistent with neuroanatomical differences in activation of proximal and distal muscles.

## Introduction

As the saying goes, practice makes perfect, and training a motor skill generally leads to improvements in performance. One of the oldest principles of motor learning is the *specificity of practice hypothesis* (Thorndike and Woodworth, 1901), proposing that training effects are highly task- and effector-specific (e.g., Sanes and Donoghue, 2000; Giboin et al., 2015; Spampinato et al., 2017). However, training often has additional learning effects that were not specifically trained. These additional learning effects are collectively labeled motor learning transfer (Adams, 1987). Transfer of skills has been subject to extensive investigation for over a century (Thorndike and Woodworth, 1901; Thorndike, 1903). Researchers traditionally define transfer of learning as the influence of previous experiences on performing the same skill in a new context or on learning a new skill (Magill and Anderson, 2014). But also within the same task performed in the same situation, learning transfer can occur from the trained effector to several other untrained effectors. For example, transfer can occur between the same lateral body parts and already in 1903, Swift showed that training the dominant hand in juggling two balls improves performance of the other non-trained hand on the same task as well (Swift, 1903). Similarly, several performance characteristics turn out to be preserved between effectors, e.g., between arms and feet (Keele et al., 1985), between limbs (Wright, 1990), between limb and oral movements (Franz et al., 1992), from hand to foot on either side and vice versa (Oxendine, 1984), and between ipsilateral and diagonal arm and leg body links (Kumar and Mandal, 2005). It has also been shown that there is substantial functional equivalence, meaning that a pattern learned with one effector system can be transferred to a completely different effector system to realize the same task (Kelso and Zanone, 2002). In addition, several studies have demonstrated that learning transfer depends on whether the dominant or non-dominant side is trained (e.g., Taylor and Heilman, 1980; Parlow and Kinsbourne, 1989; Thut et al., 1996; Yoo, 2015). However, the existing research on the pattern and direction of transfer is not consistent and seems to depend on the tasks and performance variables used in the different learning transfer studies.

Despite the well-established existence of motor learning transfer, controversy remains about the origin and mechanisms underlying this learning transfer. A possible reason for the controversy might relate to different transfer studies having used different tasks, which potentially may have given rise to different results. In order to explain and understand the mechanisms underlying positive transfer of learning, several theories and hypotheses have been put forward to account for learning transfer across different body parts, tasks, and situations from various perspectives, but a conclusive explanation of the responsible mechanisms for motor skill transfer is still unclear (for a review, see Issurin, 2013; Magill and Anderson, 2014). One of the first prominent theories of transfer was based on identical elements theory (Thorndike and Woodworth, 1901; Thorndike, 1903). The identical elements theory considers the level of similarities in skill and context components, and similarity of processing requirements (also referred to as transfer-appropriate processing) (Lee, 1988; Magill and Anderson, 2014). The higher the level of similarities, the more learning transfer is expected to occur. According to this theory, specific training has the highest level of similarity, and therefore shows superior learning effects (Sanes and Donoghue, 2000; Giboin et al., 2015; Spampinato et al., 2017).

From a motor control and learning perspective, more recent learning transfer theories have focused on cognitive functions and mental practice (e.g., Kohl and Roenker, 1980), generalized motor programs (Shapiro, 1977; Schmidt, 1982; Schmidt and Lee, 2005), coordination dynamics (Kelso et al., 1979; Kelso and Zanone, 2002), or neurophysiological explanations focusing on central and peripheral neural regulation (Hicks et al., 1983; Kristeva et al., 1991; Cramer et al., 1999; Schultze et al., 2002; Hortobagyi et al., 2003).

As mentioned above, one possible explanation for the discrepancies in different studies and theories on motor learning transfer might be related to the variety of tasks and effectors studied. From a neurophysiological perspective, the morphological differences between proximal and distal effectors are an interesting entry for further research on transfer of motor learning (e.g., Aune et al., 2013, 2015). For example, studying transfer effects in proximal and distal effectors in the upper extremities allows for the distinction between bilateral learning transfer between homologous effectors (such as learning transfer from trained right hand to untrained left hand), lateral transfer of learning between non-homologous effectors (such as learning transfer from trained right hand to untrained right shoulder), and bilateral transfer of learning between non-homologous effectors (such as learning transfer from trained right hand to untrained left shoulder). Note that lateral transfer between homologous effectors would not refer to learning transfer but to specific learning. Below, we address each of the three types of transfer more specifically.

*Bilateral learning transfer between homologous effectors* is arguably the type of learning transfer that has been investigated most intensively (Swift, 1903; Bray, 1928; Pan and Van Gemmert, 2013; Yao et al., 2014). The neurophysiological explanations of bilateral learning transfer are based on the theory of neural interaction and communication in the spinal cord (that is, peripheral neural regulation) and at the cortical level (that is, central neural regulation). The peripheral neural regulation in the complex network of circuits in the spinal cord influences motor output with both inhibitory and excitatory effects (Jankowska, 1992; Pierrot-Deseilligny and Burke, 2005), and studies of unilateral contractions or movements have shown a gain in modulation of contralateral spinal circuits (Hortobagyi et al., 2003). During unilateral actions the interneurons that receive afferent and descending inputs cross the midline to excite or inhibit contralateral motor neurons (Jankowska et al., 2005a,b) that in turn innervate whole body, axial, and proximal movements. It seems likely that these interneurons contribute to crossed effects in humans (Delwaide and Pepin, 1991) that might facilitate bilateral transfer of motor learning for proximal muscles in particular.

In addition to peripheral neural regulation, there is also neural interaction and communication at the cortical level, so-called central neural regulation. This regulation focuses on the interaction of the primary motor cortex (M1) of the two hemispheres when performing unilateral contractions (e.g., Abbruzzese et al., 1999; Taniguchi et al., 2001; Bloom and Hynd, 2005; Daffertshofer et al., 2005; Post et al., 2007). In general, unilateral muscle contractions mainly involve activation from one hemisphere, but there is also a significant interaction and bilateral communication with the contralateral hemisphere (e.g., Tettamanti et al., 2002; Omura et al., 2004; Perez et al., 2007). The two cerebral hemispheres are connected through the corpus callosum with the primary function to provide interactions between homologous cortical areas (Hellige, 1993; Bloom and Hynd, 2005). These interhemispheric interactions can have both excitatory and inhibitory effects, and can both increase and decrease neural drive to the contralateral hemisphere and muscles during unilateral contractions (Oda and Moritani, 1995; Khodiguian et al., 2003). This again is likely to influence bilateral learning transfer (Schultze et al., 2002). In primates, the number of transcallosal projections connecting proximal muscles are significantly larger compared to those for distal muscles (Pandya and Vignolo, 1971; Jenny, 1979; Gould et al., 1986; Rouiller et al., 1994; Brodal, 2004). The distal muscles are innervated through monosynaptic pathways to a larger extent (Kuypers, 1978; Palmer and Ashby, 1992) than those targeting proximal arm muscles (Brinkman and Kuypers, 1972; Aglioti et al., 1993). These morphological differences might weaken the potential for interhemispheric communication and thereby bilateral transfer of skill components for distal compared to proximal effectors.

The second type of learning transfer is between different effectors on the same body side, labeled *lateral transfer of learning between non-homologous effectors*. Such intra-limb transfer has been studied previously in writing skills (Merton, 1972; Wright, 1990), and has shown striking similarities in letter shapes between writing with the finger and wrist of the dominant hand and shoulder-elbow writing movements of the same limb. Vangheluwe et al. (2004) studied intralimb learning transfer of a drawing task between proximal and distal effectors as well. The results demonstrated intralimb transfer both from proximal to distal joints and vice versa, but proximal-to-distal transfer was larger than distal-to-proximal, which was labeled a proximal-to-distal gradient (Vangheluwe et al., 2004; Aune et al., 2015). From a neurophysiological perspective, lateral transfer requires intra-hemispheric transfer of information, therefore an explanation for the proximal-to-distal gradient might be that there are more dense ipsilateral corticospinal projections to proximal effectors causing more activation and ipsilateral transmission of information compared to distal effectors (Colebatch et al., 1991; Harrison, 1991; Mack et al., 1993; Nirkko et al., 2001).

The third type of learning transfer is from one effector on one body side to a different effector on the other side, labeled *bilateral transfer of learning between non-homologous effectors*. To the best of our knowledge, no study so far has investigated this type of motor learning transfer. This third type of motor learning transfer can be seen as an interaction between bilateral transfer of learning between homologous effectors and lateral transfer of learning between non-homologous effectors, and might provide additional understanding of underlying mechanisms of motor learning transfer. However, positive transfer and generalization has been observed between different effector systems that share common task-specific coordination dynamics (Kelso and Zanone, 2002; Buchanan, 2004).

The current study aims to advance our knowledge regarding generalizability of motor skill learning by addressing all three types of learning transfer after training of either proximal or distal effectors. Regarding bilateral transfer, we examined to what extent a task acquired with a proximal or distal effector system at one side of the body is transferred bilaterally to the same (homologous) effector system at the other side, and whether the transfer effects follow a proximal-distal gradient. We expected to find more transfer for homologous proximal effectors compared to homologous distal effectors because of their differences in peripheral and central neural interaction and communication. In addition, we examined both lateral and bilateral transfer between non-homologous effectors, where we also expected to find a proximal-to-distal gradient. Finally, we also investigated specific learning in order to confirm the superior effect of specific training compared to the transfer conditions.

## Materials and Methods

### Participants

In total 28 university students (mean age 23.1 ± 1.9 years) with no known neuromuscular disorders or functional limitations participated in this study. The participants were assigned to three groups: (1) the proximal training group (5 men and 5 women), (2) the distal training group (5 men and 5 women), and (3) the control group (CG) (4 men and 4 women). All participants were right-handed as indicated by the Edinburgh Handedness Inventory (Oldfield, 1971). All participants gave their informed consent prior to the experimental procedure. The study was evaluated and approved by the Regional Ethical Committee and performed in accordance with the Declaration of Helsinki.

### Task and Apparatus

**FIGURE 1:**
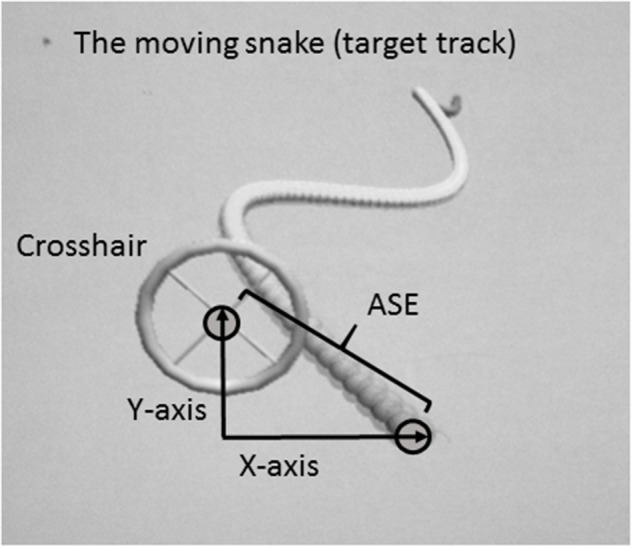
Illustration of the customized task (a 2D virtual snake) and calculation of the absolute spatial error (ASE).

A customized 2D virtual “moving snake” task was designed for the purpose of the present experiment. The moving snake task consisted of a criterion waveform made by the head of the snake and a controllable crosshair with which the participants had to track the target (head of the snake) as precisely as possible (**Figure 1**). The criterion waveform was the same in every trial. In each condition, the subjects were instructed to position the center of the crosshair at the head of the snake and follow the undulating moving snake head as closely as possible. When the center of the crosshair was perfectly located on the head of the snake, the color of the snake head changed, thereby functioning as online feedback to the subjects. The moving snake task was made using the Unity3D game engine and programmed using C#. The sampling frequency of the task was 100 Hz. For each sample the following information was stored: time since the game started, the target point’s x- and y- coordinates, and the crosshair’s x- and y-coordinates. Two different customized joysticks were used in order to dissociate and perform isolated movements of the proximal and distal effectors, a customized proximal joystick controlled by shoulder and elbow, and a customized distal joystick controlled by wrist and index finger. The joysticks were operated with both dominant and non-dominant side, in a total of four different conditions.

**FIGURE 2:**
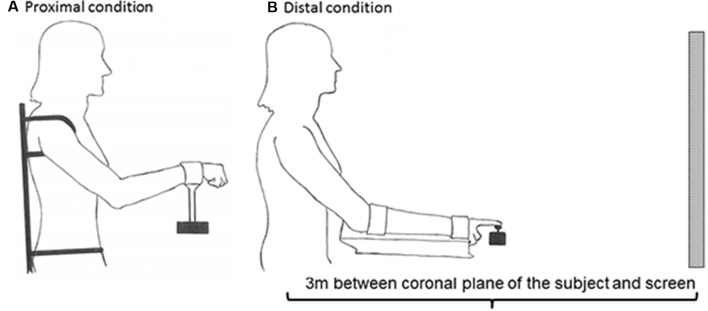
Experimental set-up. In order to prevent mechanical, postural, and synergist muscle contributions in the proximal **(A)** and distal **(B)** conditions, the participants’ body positions were constrained by clamps and straps as illustrated. The starting position in the proximal condition was calibrated to 45° between the trunk and overarm (humerus), and 130° between humerus and radius **(A)**. The starting position in the distal condition was calibrated to 25° between the trunk and overarm, with the underarm resting in a horizontal position **(B)**. The subject was positioned 3 m from the screen in both conditions.

Furthermore, a custom-made chair and apparatus were used to prevent postural instability and activation of other postural core muscles, thereby limiting activation to only shoulder-elbow in the proximal condition and only wrist-index finger in the distal condition. To ensure the use of only shoulder and elbow movements in the proximal condition, the trunk and the upper body were strapped to the chair (**Figure 2A**). In addition, the height of the seat was elevated in order to eliminate activation of the feet. In order to isolate the use of wrist and index fingers, the forearm rested on a platform to which it was strapped (**Figure 2B**). The screen had a width of 148 cm and a height of 110 cm. The amplitude of the function was adjusted to be within 1/3 of the height of the screen.

### Procedure

Each participant had eight individual sessions in the lab. On the first day, the participants were informed about the task, signed an informed consent form, and the task was demonstrated by the experimenter. The subjects were naive about the hypotheses of the study. The next day the participants completed a pre-test consisting of the following four test conditions: (1) Proximal effectors dominant side, (2) Distal effectors dominant side, (3) Proximal effectors non-dominant side and (4) Distal effectors non-dominant side.

The participants had one practice trial in each condition first in order to familiarize themselves with the apparatus, the task, and the different conditions. After the participants were accustomed to the task and each condition, baseline performance in the different conditions was captured (pre-test). Each of the four conditions was performed three times, resulting in a total of twelve trials. The order of the respective conditions was counterbalanced across participants.

After the pre-test, the participants were divided into three groups based on their mean scores on the four pre-test conditions. The criterion score for each participant was calculated by summarizing mean performance score from each pre-test condition, and divided by the number of conditions (4 conditions). The Proximal Effector Training Group (PETG) controlled the joystick during the training period with dominant shoulder-elbow joints, while the Distal Effector Training Group (DETG) controlled the joystick with dominant wrist-index finger. The CG did not receive training and only performed the pre- and post-tests.

The two training groups trained on five consecutive days, with each daily session consisting of 25 trials of 30 s each. Each training session was subdivided in 5 blocks with five trials each, followed by a 2 min rest period in order to maintain motivation and prevent fatigue. At the end of each training session the experimenter provided verbal feedback, accompanied by a visual graphic feedback of the last trial on the screen in front of the participants. The CG did not practice between the pre-test and post-test. The day after the last training session for the experimental groups, the post-test was conducted on all three groups. The post-test was identical to the pre-test except for the initial practice trials.

### Data Analysis

The dependent variable was the average Absolute Spatial Error (ASE) between the target and the crosshair. The unit of measurement was in virtual meters as defined in the customized software. ASE was measured as the distance between the head of the snake and the crosshair, calculated by the Pythagoras equation (**Figure 1**):

$$\text{Absolute Spatial Error }\left(\text{ASE}\right)=\sqrt{\left(x^{2}+y^{2}\right)}$$

All experimental testing was conducted over a 30 s test period with a sampling frequency of 100 Hz, in total 3000 samples. In order to analyze steady state performance only the samples from 300 to 2700 were analyzed, in total 2400 samples in each condition. The first and the last 300 samples were excluded because the first samples could be influenced by tuning in to the experimental task, while the last were excluded to avoid of loss of concentration, fatigue, or mobilizing extra effort (e.g., Repp and Penel, 2004; Moe-Nilssen and Helbostad, 2005; Lorås et al., 2012). The average across three repeated trials was calculated and used in further analyses.

Movement control has previously been shown to be more accurate on the dominant side as well as in distal compared to proximal joints (Aune et al., 2015). Therefore, we calculated the relative improvement in ASE for each of the four test conditions according to the following equation:

$$\text{Relative Improvement }\left(\Delta \text{ASE Index}\right)={\left((\text{ASE Pretest}-\text{ASE Posttest})/\text{ASE Pretest}\right)}^{*}100\%$$

A ΔASE Index of 0% means no change from pre- to post-test, a ΔASE Index of 50% indicates that the post-test error is half the size of the pre-test error, and 100% indicates perfect performance (no error) on the post-test.

### Statistical Analysis

Dependent variables were the ASE and relative improvement between pre- and post-test (ΔASE index) for the four effectors (left proximal and distal, right proximal and distal). All variables were normally distributed, as indicated by Kolmogorov–Smirnov tests. To test equality between the three groups at pre-test, a one-way ANOVA Group (PETG, DETG, control) was performed on ASE for each of the four conditions.

In order to test performance differences between pre- and post-test in the three groups in the four conditions, paired-samples *t*-tests were performed on ASE.

To test potential pre-existing effector and side differences, a two-way repeated measures ANOVA Effector (Proximal, Distal) by Side Dominance (Preferred side, Non-Preferred side) was performed on pre-test ASE.

To evaluate potential differences in learning conditions between the proximal and distal training groups, a two-way repeated measures mixed model ANOVA on Training Group (2) by Learning Type (4) was performed on ΔASE index. As described above, the four learning types were Specific learning, and three learning transfer conditions: Bilateral learning transfer between homologous effectors, Lateral learning transfer between non-homologous effectors, and Bilateral learning transfer between non-homologous effectors. *Post hoc* follow-up of significant effects consisted of pairwise comparisons with Bonferroni corrections for multiple comparisons.

All statistical analyses were performed in SPSS (Version 23.0, SPSS, Inc., Chicago, IL, United States), and a criterion alpha level of *p* < 0.05 was used for statistical significance.

## Results

Descriptive analyses of the ASE at pre- and post-test are presented in **Table 1**. A one-way ANOVA confirmed that the three groups did not differ in ASE at pre-test in any of the conditions (all *p*’s > 0.326).

**Table 1:** Mean and SD of Absolute Spatial Error on pre- and post-test for all conditions for the distal (DETG) and proximal (PETG) training groups and the control group (CG).

		Distal dominant		Proximal dominant		Distal Non-dominant		Proximal Non-dominant	
					
		*Pre*	*Post*	*Pre*	*Post*	*Pre*	*Post*	*Pre*	*Post*
DETG	Mean	0.791	0.474	0.969	0.822	0.802	0.650	0.996	0.952
	*SD*	0.164	0.075	0.108	0.120	0.127	0.078	0.153	0.167
PETG	Mean	0.724	0.517	0.862	0.516	0.750	0.617	0.968	0.673
	*SD*	0.063	0.072	0.194	0.118	0.067	0.099	0.159	0.124
CG	Mean	0.760	0.722	0.900	0.852	0.818	0.801	0.989	0.946
	*SD*	0.175	0.080	0.193	0.134	0.102	0.120	0.115	0.131


Performance improvement between pre- and post-tests was tested by paired-samples *t*-tests on ASE. PETG had significant improvement from pre- to post-test in all four conditions (all *p*’s < 0.001), while there was significant improvement in three out of four conditions for the distal training group (all *p*’s < 0.001 except for bilateral transfer between non-homologous effectors, *p* = 0.09). No significant differences in performance between pre- and post-test were shown in any of the conditions for the CG (all *p*’s > 0.20).

### Effector and Side Differences

To test for potential pre-existing differences between effectors and side-dominance, a two-way repeated measures ANOVA Effector (Proximal, Distal) by Side Dominance (Preferred side, Non-Preferred side) was performed on pre-test ASE. It confirmed a main effect for both Effector [*F*(1,27) = 62.53, *p* < 0.0005] and Side Dominance [*F*(1,27) = 11.32, *p* < 0.005], confirming that the ASE is significantly less for distal effectors compared to proximal effectors, and less for the preferred side compared to the non-preferred side. Therefore, the relative ASE, ΔASE index, will be used below to investigate potential differences between the different conditions in the amount of learning.

### Amount of Specific Learning and Learning Transfer

**FIGURE 3:**
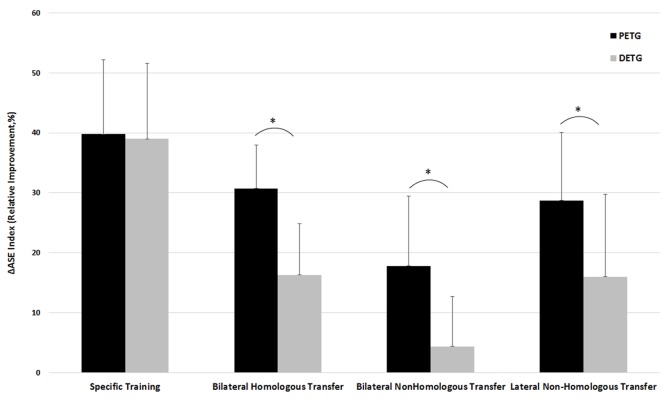
Comparison of the training effect for PETG (Proximal Effector Training Group) versus DETG (Distal Effector Training Group) measured as ΔASE index for the conditions Specific learning, Bilateral transfer between homologous effectors, Lateral (intra-limb) transfer between non-homologous limbs, and Bilateral transfer between non-homologous limbs. ^∗^Indicates a significant difference in training effect between PETG and DETG.

Significant learning effects were found for 7 out of 8 comparisons in the two training groups, but the amount of learning might differ between the different types of learning for the proximal versus distal training groups. To test for potential differences in learning transfer effects for the training groups, a two-way repeated measures mixed model ANOVA on Training Group (PETG, DETG) by Learning Type (4: Specific learning, Bilateral learning transfer between homologous effectors, Lateral learning transfer between non-homologous effectors, and Bilateral learning transfer between non-homologous effectors) was performed on ΔASE index. It confirmed a main effect for Training Group on ΔASE index [*F*(1,18) = 10.15, *p* < 0.005] and a main effect of Learning Type [*F*(3,16) = 35.93, *p* < 0.0005]. Pairwise comparisons between the four learning types with Bonferroni corrections indicated that specific learning was significantly more effective compared to the three transfer types, that bilateral learning transfer between homologous effectors was not significantly different from bilateral learning transfer between non-homologous effectors, and that lateral learning transfer between non-homologous effectors was significantly less effective than the other types (all *p*’s < 0.002, see **Figure 3**).

In addition, there was a significant Training Group by Learning Type interaction [*F*(3,16) = 4.52, *p* < 0.02], indicating that the ΔASE index may be larger for PETG than for DETG in some conditions but not all. The interaction was followed up by *post hoc* independent samples *t*-tests on each condition, confirming that the proximal training group showed larger learning effects than the distal training group in all learning types (all *p*’s < 0.01) except for specific learning where both groups performed equal (**Figure 3**). Below, we describe in more detail the effects of specific training and the three transfer conditions.

### Specific Learning

For the specific learning type, training occurred on the same effector as tested in the post-test condition. Training of both proximal and distal effectors resulted in large improvements of the ΔASE index, namely 39.77 ± 12.44% and 38.97 ± 12.61%, respectively (**Figure 3**). The *post hoc* follow-up of the main effect of Learning Type described above showed that specific learning was more effective than all three types of learning transfer, all *p*’s < 0.0005. There was no significant difference between the proximal and distal training groups regarding specific learning, *t*(18) = -0.143, *p* = 0.89.

### Bilateral Learning Transfer between Homologous Effectors

Learning transfer to the contralateral homologous effector systems was demonstrated for both proximal and distal effectors. Improvement of the ΔASE index was 30.70 ± 7.31% and 16.34 ± 8.46%, respectively. The *post hoc* follow-up of Learning Type showed that bilateral learning transfer between homologous effectors was less effective compared to specific learning (*p* < 0.0005), but significantly more effective compared to bilateral learning transfer to the non-homologous effectors (*p* = 0.001). No differences were shown for bilateral learning transfer between homologous effectors versus lateral learning transfer between non-homologous (*p* = 1.000). An independent samples *t*-test on ΔASE index showed significantly larger bilateral learning transfer to the contralateral homologous effectors for the proximal versus distal training group, *t*(18) = -4.062, *p* < 0.001 (**Figure 3**).

### Lateral Learning Transfer between Non-homologous Effectors

Lateral learning transfer between non-homologous effectors was demonstrated for both proximal and distal effectors, with improvements of the ΔASE index being 28.68 ± 11.40%, and 14.02 ± 8.54%, respectively. The *post hoc* follow-up of Learning Type showed that lateral learning transfer between non-homologous effectors was less effective compared to specific learning (*p* < 0.0005), but more effective compared to bilateral learning transfer between non-homologous effectors (*p* = 0.002). No differences were shown for lateral learning transfer between non-homologous effectors and bilateral learning transfer between homologous effectors (*p* = 1.000). An independent samples *t*-test on ΔASE index showed significantly larger lateral learning transfer between non-homologous effectors for the proximal versus distal training group, *t*(18) = -3.254, *p* = 0.004 (**Figure 3**).

### Bilateral Learning Transfer between Non-homologous Effectors

Training with the dominant proximal effector resulted in significant bilateral learning transfer to the distal non-homologous effector, while training with the dominant distal effector system did not show significant bilateral learning transfer to the proximal non-homologous effector. Improvement of the ΔASE index was 17.78 ± 11.55% and 4.36 ± 8.27%, respectively. The *post hoc* follow-up of Learning Type showed that bilateral learning transfer between non-homologous effectors was the least effective compared to the other three learning types, all *p*-values < 0.002. An independent samples *t*-test on ΔASE index showed significantly larger bilateral learning transfer between non-homologous effectors for the proximal versus distal training group, *t*(18) = 2.988, *p* = 0.008 (**Figure 3**).

## Discussion

The main purpose of the current study was to elucidate potential differences in learning transfer after training proximal versus distal effectors in the upper extremities. In addition, we investigated specific learning effects in the same effectors. The theoretical background for the study was derived from motor learning theories about learning transfer combined with models of morphological and functional differences between proximal and distal effectors. In line with the theories of motor learning and learning transfer, it was hypothesized that specific training would be the most effective type of training irrespective of trained effector. Furthermore, grounded in the models of neural communication and transmission of motor control, we expected to find a proximal-distal gradient in motor learning transfer. The results confirmed specific training to be the most effective for performance improvement for both proximal and distal effectors, and demonstrated a general transfer and thus generalizability of performance when training with both proximal and distal effectors to other non-trained effectors in the upper extremities. Most importantly, the results supported our hypothesis of a proximal-distal gradient in motor learning transfer, in that we found more pronounced learning transfer to non-trained effectors in upper extremities after training with proximal effectors versus training with distal effectors. No significant improvement was shown for the non-training CG in any of the conditions.

### Specific Learning

The superior effect of specific motor learning for both the proximal and distal training groups confirms that learning effects are highly specific to the particular task trained, as proposed by the specificity of learning hypothesis (Henry, 1958, 1968), and these findings are in line with earlier research (e.g., Henry, 1958; Grafton et al., 1998; Giboin et al., 2015). There was no significant difference between the proximal and distal training groups in specific motor learning, indicating that training elicits specific neural adaptations to the motor system irrespective of the effector trained (Edelman, 1992; Schubert et al., 2008). In addition to the superior effect of specific motor training, the present study also found a general positive learning transfer of motor skills to untrained effectors. This indicates that learning effects are partly effector independent and can generalizable to other effectors.

### Bilateral Learning Transfer between Homologous Effectors

The most interesting transfer effect explored in the present study was the potential difference in bilateral motor learning transfer between proximal and distal homologous effectors. Based on the theory of bilateral transmission and communication between hemispheres and in the spinal cord, we expected to find more pronounced bilateral motor learning transfer for proximal versus distal homologous effectors. A behavioral approach was selected to study how unilaterally practiced upper limb skills transfer to the performance of the same effector on the opposite, untrained side. Behavioral studies of bilateral transfer have shown consistency of this phenomenon across both homogenous proximal and distal effectors. Already in 1903, Swift conducted a study of bilateral transfer for gross motor skills (proximal effectors), while Cook (1934) and Baker et al. (1950) demonstrated the effect of bilateral transfer for fine motor skills (distal effectors). The results of the present study are consistent with these earlier findings, and document that a task acquired with an effector system at one side of the body is partly bilaterally transferred to the homologous effector system at the other side for both the proximal and the distal training groups.

The morphological differences in transmission and communication between hemispheres and in the spinal cord for proximal and distal muscles are well documented (e.g., Elliot et al., 1999; Beaton, 2004), but the potential behavioral effects of these differences on bilateral communication and motor control of proximal and distal effectors has received less attention. These morphological differences prompted us to investigate the hypothesis that bilateral learning transfer should be larger for proximal than for distal homologous effectors. To the best of our knowledge, the current study is the first to explicitly examine and compare the magnitude of bilateral transfer of learning effects for proximal versus distal effectors. The results of the present experiment showed that bilateral transfer of learning effects between homologous proximal and distal effectors is indeed asymmetrical, with larger bilateral proximal to proximal transfer than bilateral distal to distal transfer. This confirms our hypothesized proximal-distal gradient in bilateral learning transfer between homologous effectors, which is assumed to be mediated by differences in interhemispheric transmission of information via the pathway of commissural fibers in the corpus callosum and interneurons between the lamina VIII in the spinal cord (Lassonde, 1986; Hortobagyi et al., 2003; Jankowska et al., 2005b). The differences in potential for bilateral communication for proximal and distal effectors seem to be important for – and have a functional effect on – bilateral motor learning transfer, dependent on the effectors involved.

### Lateral Learning Transfer between Non-homologous Effectors

In addition to the bilateral learning transfer between homologous effectors described above, the current results revealed positive lateral learning transfer between non-homologous effectors (indicating intra-limb transmission of learning effects) for both the proximal and distal effectors. These results are consistent with previous unimanual and bimanual transfer studies (e.g., Merton, 1972; Wright, 1990). Interestingly, the results indicated that intra-limb transfer is asymmetrical for proximal and distal effectors as well, with better proximal-to-distal transfer than vice versa. These results partly replicate findings of earlier studies of drawing tasks that revealed performance improvement for intermanual training for proximal but not distal effectors (Thut et al., 1996; Vangheluwe et al., 2004). From a neurophysiological perspective, such lateral transfer requires intra-hemispheric transmission of information, and an explanation for the proximal-distal gradient might be that proximal effectors have more dense ipsilateral corticospinal projections compared to distal effectors (Colebatch et al., 1991; Harrison, 1991; Mack et al., 1993; Nirkko et al., 2001).

### Bilateral Learning Transfer between Non-homologous Effectors

Transfer of learning effects to inter-limb non-homologous effectors was shown for dominant proximal effectors to non-dominant distal effectors, but not for dominant distal effectors to non-dominant proximal effectors. These results are consistent with what earlier studies demonstrated in unimanual skills such as writing and drawing (Hicks, 1974; Wright, 1990; Imamizu et al., 1998; Morton et al., 2001), and are in congruence with transfer to be more effective from large (e.g., proximal effectors) to smaller (e.g., distal effectors) scale movements (Wilde and Shea, 2006; Dean et al., 2008). These results show that motor learning is at least partly generalizable, and indicate that memory of movement to some extent might be abstract and effector-independent (Vangheluwe et al., 2004).

## General Discussion and Conclusion

As the results of the current study illustrate, specific training of effectors is superior to transfer of learning effects to untrained effectors, confirming that motor control is characterized by a relatively high level of neuromuscular specificity. More interestingly, the transfer or generalizability of learning effects is larger for proximal effectors compared to distal effectors, which is consistent with the neuroanatomical differences between proximal and distal effectors. These results pave the way for further studies that combine a behavioral study with direct measures of neural activation to illuminate the potential link of communication and transfer of learning in motor performance between different effectors in general, and potential differences between proximal and distal effectors in particular.

The functional and behavioral data from the current study do not evaluate the predictions of transmission of information directly, but encourage further research to include measures both of brain activity, e.g., electroencephalography (EEG) or functional magnetic resonance imaging (fMRI), and of muscle activity measures through, e.g., electromyography (EMG). Such measures could provide additional insights into the interhemispheric communication of proximal and distal effectors and the potential effects on transfer of learning. They can also illuminate whether learning transfer is caused by interhemispheric interactions alone, or whether spinal contributions should be considered in addition. Similarly, brain activity measurements can elucidate to what extent learning transfer has a neural origin, or whether more general principles for learning need to be taken into account. Incorporating such measures could give further and more detailed information about the differences in learning transfer for proximal versus distal effectors. The present study does not evaluate potential asymmetries of transfer after practicing the non-dominant proximal and distal effectors. It would be an interesting follow-up study to examine potential asymmetries in the direction of transfer effects when training with dominant versus non-dominant proximal and distal effectors.

To conclude, the current study contributes to our understanding of motor control and learning processes as it addresses both the effect of specific training of effector systems and learning transfer to different effector systems. It was hypothesized that the morphological differences between proximal and distal effectors would result in more pronounced transfer of learning in proximal compared to distal effectors, which was confirmed by the observed proximal-distal gradient in our results. As such, the study informs about potential neuromuscular constraints for the motor control system of proximal and distal effectors, the possibilities and limitations regarding transfer of motor learning capabilities in general, and the differences in transfer between proximal and distal effector systems in upper extremities in particular.

## Ethics Statement

This study was evaluated by and carried out in accordance with the recommendations of the Norwegian regional ethical committee. All subjects gave written informed consent in accordance with the Declaration of Helsinki.

## Author Contributions

All authors listed have made a substantial, direct and intellectual contribution to the work, and approved it for publication.

## Conflict of Interest Statement

The authors declare that the research was conducted in the absence of any commercial or financial relationships that could be construed as a potential conflict of interest.
